# Comparison of fMRI Digit Representations of the Dominant and Non-dominant Hand in the Human Primary Somatosensory Cortex

**DOI:** 10.3389/fnhum.2018.00492

**Published:** 2018-12-10

**Authors:** Meike A. Schweisfurth, Jens Frahm, Dario Farina, Renate Schweizer

**Affiliations:** ^1^Biomedizinische NMR, Max-Planck-Institut für biophysikalische Chemie, Göttingen, Germany; ^2^Fakultät Life Sciences, Hochschule für Angewandte Wissenschaften Hamburg, Hamburg, Germany; ^3^Department of Bioengineering, Imperial College London, Royal School of Mines, London, United Kingdom; ^4^Leibniz-ScienceCampus Primate Cognition, Göttingen, Germany

**Keywords:** somatotopy, digits, primary somatosensory cortex, fMRI, left, right

## Abstract

The tactile digit representations in the primary somatosensory cortex have so far been mapped for either the left or the right hand. This study localized all ten digit representations in right-handed subjects and compared them within and across the left and right hands to assess potential differences in the functional organization of the digit map between hands and in the structural organization between hemispheres. Functional magnetic resonance imaging of tactile stimulation of each fingertip in BA 3b confirmed the expected lateral-anterior-inferior to medial-posterior-superior succession from thumb to little-finger representation, located in the post-central gyrus opposite to the motor hand knob. While the more functionally related measures, such as the extent and strength of activation as well as the Euclidean distance between neighboring digit representations, showed significant differences between the digits, no side difference was detected: the layout of the functional digit-representation map did not consistently differ between the left, non-dominant, and the right, dominant hand. Comparing the absolute spatial coordinates also revealed a significant difference for the digits, but not between the left and right hand digits. Estimating the individual subject's digit coordinates of one hand by within-subject mirroring of the other-hand digit coordinates across hemispheres yielded a larger estimation error distance than using averaged across-subjects coordinates from within the same hemisphere. However, both methods should only be used with care for single-subject clinical evaluation, as an average estimation error of around 9 mm was observed, being slightly higher than the average distance between neighboring digits.

## Introduction

From Penfield's seminal studies (Penfield and Boldrey, 1937; Penfield and Rasmussen, 1950), it is well-known that the primary somatosensory cortex (SI) at the post-central gyrus has a somatotopic map of the body. Functional magnetic resonance imaging (fMRI) studies, using tactile stimulation at the first phalanx of the digits, have investigated the locations of digit representations within one hand in greater detail (Kurth et al., 1998; Nelson and Chen, 2008; Schweizer et al., 2008; Sanchez-Panchuelo et al., 2010, 2012; Schweisfurth et al., 2011, 2014, 2015; Besle et al., 2013; Martuzzi et al., 2014). A consistent sequence of digit representations could be shown in the contralateral primary somatosensory cortex, starting with the little finger (D5) in a superior-medial-posterior position, and proceeding with the ring finger (D4), the middle finger (D3), the index finger (D2), and the thumb (D1) in the inferior-lateral-anterior direction along the central sulcus.

This sequence of functional somatosensory digit activation is closely linked to a landmark structure in the upper third of the central sulcus, the superior genu, a posterior bend of the precentral gyrus (Broca, 1888). This structure has been shown to have a close association with the functional motor representation of the hand in the precentral-gyrus motor cortex and is therefore named “hand knob,” (Yousry et al., 1997). Based on the tight link between the hand/finger representation in the motor cortex and the finger representation in the somatosensory cortex, the hand knob is correspondingly a structural landmark for the finger representations in the opposite post-central gyrus (Schweizer et al., 2008; Schweisfurth et al., 2011, 2014, 2015).

The general principle of D1-to-D5 succession of functional activation in the contralateral hand-knob SI area holds for both hands. While former studies have mapped either the fingers of the right, mostly dominant hand (Kurth et al., 1998; Nelson and Chen, 2008; Schweizer et al., 2008; Schweisfurth et al., 2011, 2014, 2015; Martuzzi et al., 2014), or the left hand (Sanchez-Panchuelo et al., 2010, 2012; Besle et al., 2013; Schweisfurth et al., 2015), the present study realizes the direct within-subject comparison of the digit representations of the right, dominant, with the left, non-dominant hand by mapping all ten fingertips in each subject. BOLD activation, elicited by tactile stimulation of each of the five fingers in each hand, was determined in contralateral BA 3b, the primary tactile thalamic input area of SI, and several parameters were obtained to describe the similarities and dissimilarities in the functional and structural aspects of the digit representations within the contralateral hemispheres.

One aim of the study was to investigate if the difference in hand use between the dominant and the non-dominant hand in right handers would be reflected in the tactile somatosensory digit representations. For this purpose, mainly functionally related measures such as strength and extent of the elicited BOLD activations were compared between the digits as well as between the dominant right and the non-dominant left side. Since the somatosensory system is well known for the plasticity in the functional maps of the digit representations in reaction to changes in peripheral tactile input to the fingers (Recanzone et al., 1992; Elbert et al., 1994; Sterr et al., 1998; Braun et al., 2000), the more extended and differentiated motor use of the dominant hand might also influence the general spatial relationship between neighboring digits in the tactile somatosensory system. Therefore, the relative Euclidean distances between each single-digit representation and its next neighboring digit representation were obtained within the left and right hands and compared between sides to explore potential differences.

A second aspect of the study was to explore and compare the absolute spatial position of the digit representations. The absolute positions in x-, y-, z- coordinates reflect the combination of functional and structural aspects influencing the layout of the digit maps. The structural layout of the hand knob area puts certain constrains on the positions of the digit representations along the post-central gyrus, and the spatial location of the digit representations can, within these limits, vary in response to functional aspects such as finger usage or loss / changes in somatosensory input. Determining the coordinates of the vertices with the highest BOLD activation along the medial-lateral (x-axis), anterior-posterior (y-axis) and inferior-superior (z-axis) dimension, and flipping the finger representations in the left hemisphere onto the right hemisphere, allowed to examine, if the single digits of the two hands were in comparable locations in the two hemispheres. Since the general layout of the brain and its main sulci, here in particular the central sulcus, is symmetrical, no major structural difference between the two hemispheres would be expected. But on the more detailed spatial scale of the 20 mm of the hand-knob area, hemispheric variations in the extent and wideness of the post-central gyrus windings between hemispheres can be observed in each brain. This hemispheric difference could potentially lead to divergent absolute spatial position of the homologous digit representations, when directly comparing left- and right-hand digit representations in individuals. Additionally to the absolute position within the coordinate system, the relative Euclidean distances between the D1 and the successive digit representations were obtained for each hemisphere. This measure is, at least for the longer distances to D4 and D5, influenced by the structural layout of the hand knob.

Complementary to the general questions outlined above, we also specifically calculated and analyzed the individual subjects deviations between the position of a single-digit representation and the homologous other hand's digit, again flipped from the opposite hemisphere across the interhemispheric midline. This individual “mirroring” approach estimates the spatial position of the individual representation of a digit in one hemisphere from the position of the subject's homologous digit in the other hemisphere. Clinically, this could be relevant in cases where the location of a digit representation is not measurable, such as in unilateral transradial or transhumeral amputees where the location of the remaining hand's digit representation in the other hemisphere is mirrored and used as an estimate of the other-side's digit representation prior to amputation. This builds on the original approach of the early studies of remapping in transradial amputees, in which the shift of adjacent representational areas, such as the chin, was mirrored from the affected to the non-affected hemisphere (Elbert et al., 1994; Yang et al., 1994; Flor et al., 1995). The here described analysis complements the analysis above: Even if no across-subjects consistent difference, i.e. a shift into one particular direction, was found between fingers of the two hands, it could still be that the left and right representation positions of a digit differed substantially within subjects, resulting in a large estimation error. Based on the results of the present study, also an alternative approach using the average of all other subjects' digit positions from the same hemisphere is proposed to estimate the location of single digit representations.

## Materials and Methods

### MRI

Twelve healthy subjects (5 women, range 22–35 years, mean 27.6 ± 3.9 years) took part in one or two MRI sessions. The data presented here were used as localizer runs of two other studies (Schweisfurth et al., 2014, 2015) that were approved by the ethics committee of the Georg-Elias-Müller-Institute for Psychology, Göttingen, Germany. Before each study, informed written consent was obtained from the subjects. All subjects were right-handed according to the Edinburgh Inventory [laterality index 81 ± 13, (Oldfield, 1971)].

For MRI, a 3 T system (TIM Trio, Siemens Healthcare, Erlangen, Germany) along with a 32-channel head coil was used. For all subjects, a whole-brain anatomical MR image was obtained. In addition, partial-volume fMRI data were acquired for mapping the contralateral fingertips in BA 3b. For registration and vessel identification, a whole-brain echo-planar image (EPI) and a MR angiogram were also measured. These measurements were acquired in a single session for three subjects and in two sessions for the remaining nine subjects.

The anatomical MR image was obtained by sagittal T1-weighted 3D MPRAGE (magnetization-prepared rapid gradient-echo) imaging (repetition time TR = 2,530 ms, echo time TE = 3.4 ms, flip angle = 7°, acquisition matrix = 256 × 256, 160–192 partitions, resolution = 1 × 1 × 1 mm^3^, total acquisition time TA = 10:49 min). These images were used for motor hand-knob identification at the precentral gyrus (Yousry et al., 1997) as well as for reconstruction of the surface of the white-to-gray matter border.

For fMRI mapping, a gradient-echo EPI sequence with 1.5 × 1.5 × 1.5 mm^3^ resolution was recorded (TR = 2,000 ms, TE = 36 ms, flip angle = 70°, acquisition matrix = 128 × 128, field of view = 192 × 192 mm^2^, partial Fourier factor = 6/8). For this purpose, 19 double-oblique (transverse-to-sagittal and transverse-to-coronal) sections were positioned over the hemisphere contralateral to the stimulated digits. Sections were centered around the motor hand knob and oriented perpendicular to the walls of the central sulcus, cutting it from the surface of the pre- and post-central gyri to the fundus of the central sulcus.

Subsequently, a single gradient-echo EPI volume was acquired for each hemisphere, with the same slice orientation and position of fMRI data, but covering the entire brain (81 sections, 1.5 × 1.5 × 1.5 mm^3^ resolution, TR = 8,600 ms, TE = 36 ms, flip angle = 70°, acquisition matrix = 128 × 128, field of view = 192 × 192 mm^2^, partial Fourier factor = 6/8). This greater volume was used as intermediate registration step between the functional partial-volume measurements and the anatomical whole-brain image. MR angiograms for each hemisphere were acquired with a T1-weighted 3D FLASH sequence (TR = 22 ms, TE = 4.43 ms, flip angle = 18°, resolution = 0.3 × 0.3 × 0.5 mm^3^, 57 sections from two overlapping slabs) that agreed with the hemisphere's functional partial-volume data both in volume coverage and slice orientation.

### Tactile Stimulation and fMRI Mapping

**Figure 1:**
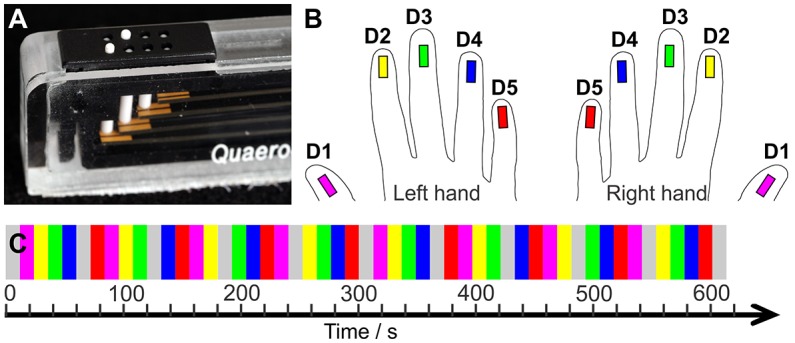
**(A)** Tactile stimulation module. **(B)** Color code: Magenta = D1, yellow = D2, green = D3, blue = D4, red = D5. **(C)** Sequential stimulation protocol. The two hands' digits were stimulated in separate runs.

Tactile stimulation of the fingertips was conducted with a piezo-electric stimulation device (QuaeroSys, St. Johann, Germany) which comprised 5 independent stimulation modules. Each module was equipped with an 8-dot Braille display (2 × 4 matrix, 2.5 × 7.5 mm^2^) at the upper side (Figure 1A). The stimulation was delivered by maximum elevation (1.4 mm if no force applied on top) of two randomly chosen pins in each stimulation cycle (square wave, stimulation interval = 10.4 ms, inter-stimulus interval = 20.8 ms, frequency = 32 Hz). Thereby, a fast-varying stimulation pattern was produced across the entire Braille display, in which the randomly chosen pins reduced the spatial adaptation and, combined with the high stimulation frequency, ensured a percept of a salient stimulation across the entire display. The 5 stimulation modules were each individually positioned below the center of the 5 respective fingertips, with the long display axis in parallel to the fingers.

The applied stimulation started with the thumb (D1) and moved sequentially across each other digit to the little finger (D5). Then, it restarted at D1. Non-stimulation rest periods were interspersed after the stimulation of four fingertips resulting in the following stimulation cycle: “D1, D2, D3, D4, Rest, D5, D1, D2, D3, Rest, D4, D5, D1, D2, Rest, D3, D4, D5, D1, Rest, D2, D3, D4, D5, Rest”. Each stimulation and non-stimulation period lasted 12 s (6 images). One mapping run, including a 20 s (10 images) baseline and two stimulation cycles, lasted 10:20 min (310 images). Across the run each digit was stimulated 8 times (96 s, 48 images) and the 20 non-stimulation rest periods occurred twice before and twice after each digit, reducing potential carry-over effects of the sequential stimulation to the neighboring digit.

Throughout the functional run, the subjects were instructed to keep their stimulated hand relaxed and pronated, without applying active pressure onto the stimulation modules. Because attended touch results in a greater fMRI SI signal than unattended touch (Johansen-Berg et al., 2000), an attentional, stimulation-dependent counting task was requested for optimizing the cortical activation strength. The subjects were asked to count covertly short, randomly-presented interruptions within the otherwise continuous stimulation (duration 156 ms, repetition every 0.5-3 s). After the run, the experimenter provided a feedback on the counting precision.

### Preprocessing And Co-Registration

BrainVoyager QX 2.6 was the main pre-processing and analysis platform used (Goebel et al., 2006) (Brain Innovation, Maastricht, The Netherlands). First, the anatomical T1-weighted images were processed. They were corrected for intensity inhomogeneity and brought into AC-PC space, i.e., the axial slice orientation was modified to be parallel to the AC-PC plane, cutting the anterior and posterior commissures and ensuring that the center of the x-axis was located along the anterior and posterior commissures, i.e., along the interhemispheric cleft. This procedure ensured that all brains comprised with the same overall orientation in 3D space, even though no stereotactic transformation was applied in order to preserve the individual size and anatomy. The resulting images were used to calculate the cortical meshes (at the white-matter gray-matter border) for the two hemispheres of each subject. Next, the functional measurements were preprocessed, including motion correction both in k-space (Siemens Healthcare, Erlangen, Germany) and in image space (BrainVoyager), followed by temporal high-pass filtering. Then, each individual functional measurement was interpolated from 1.5 mm to 1 mm isotropic spatial resolution and automatically registered to the subjects' individual anatomical AC-PC oriented images (6 degrees of freedom, 1 mm isotropic resolution, trilinear interpolation). Slight manual modifications ensured optimal registration specifically in the proximity of the motor hand knob.

FSL (FMRIB Software Library) (Smith et al., 2004) was used to register the MR angiograms of the two hemispheres concurrently to the subjects' anatomical T1-weighted image. The registration was performed with FLIRT (FMRIB's Linear Image Registration Tool, Jenkinson and Smith, 2001) via intermediate registration steps given by the first volume of the respective functional measurement and whole-brain EPI volume (see Schweisfurth et al., 2014 for further details).

The angiograms were then used to exclude vertices in the proximity of prominent blood vessels. This approach is often applied in high-resolution imaging studies of the visual system (Cheng et al., 2001; Yacoub et al., 2001; Shmuel et al., 2007) for better spatial specificity of the hemodynamic response (Polimeni et al., 2010). A previously described approach (Schweisfurth et al., 2014, 2015) for vessel exclusion in SI was used in the present study. A visual search was performed for clusters with co-located fingertip activation, defined as clusters with local peak vertices of three or more fingertips, < 3 mm apart. Because these pronounced fingertip-activation co-locations likely result from an underlying vessel, the respective angiogram was visually explored for the presence of a vessel at the cluster positions. The multiple-fingertip activation area was excluded from further analyses if a vessel was identified.

### Data Analysis

Functional activations resulting from digit stimulation were analyzed with the general linear model (GLM) including 5 predictors, one for each digit. Since the digits of the two hands were measured in different runs, they were analyzed separately. The hemodynamic response was taken into account by convolving the predictors with a two-gamma function (Friston et al., 1998). Voxels with a *t*-value greater than the threshold of false discovery rate of q(FDR) = 0.05 were assigned significant. Only the main effects for the predictors for each digit were considered, no contrast against the other digits was applied; hence, the activations of the different fingers were not contrasted against each other.

For further analysis, the functional activations upon stimulation were projected onto the individual contralateral white-matter gray-matter surface reconstruction, When creating the surface maps in Brainvoyager, the statistical information from volume space was integrated along the vertex normals, taking into account -1 to 3 mm in depth of the volume. On that surface, the analysis was restricted to putative BA 3b, defined as the posterior wall of the central sulcus. This definition by Geyer et al. (1999) is generally used in mapping studies (Nelson and Chen, 2008; Schweisfurth et al., 2011; Stringer et al., 2011), as the *in-vivo* identification of BA 3b borders is not yet possible. In the present study, the posterior bank of the central sulcus was manually outlined by including the area between the fundus of the central sulcus at the anterior end and the beginning of the curve of the post-central gyrus' crest at the posterior end (Moore et al., 2000). In the mediolateral dimension, the area comprising the activation for any of the 5 contralateral fingertips was included, plus ~1 cm at the medial and lateral end. For each of the two hemispheres, we thereby defined the individual fingertip area in BA 3b. The 3D-coordinates of the mesh-spanning vertices in these areas along with the respective thresholded *t*-values were then exported to MATLAB (MathWorks) and used for analysis.

In MATLAB, first the extent of each activation was identified for each digit representation, by counting the number of 1 mm^3^ cubes in which one or more significant vertices were found. This measure is referred to as activation volume throughout the paper. The peak vertex, being the one with the greatest significant *t*-value, was identified for each fingertip. Peak vertices are generally used to describe across-digit maps in human subjects (Kurth et al., 2000; Nelson et al., 2004; Schweizer et al., 2008). Within each side the Euclidean distances from the D2, D3, D4, and D5 peak vertices to the D1 peak vertex were calculated by $\sqrt{{\left(x_{i}-x_{1}\right)}^{2}+{\left(y_{i}-y_{1}\right)}^{2}+{\left(z_{i}-z_{1}\right)}^{2}}$, with *x*_*i*_, *y*_*i*_, and *z*_*i*_ being medial-lateral, anterior-posterior, and superior-inferior peak-vertex coordinates obtained for the *i*th finger. Similarly, the Euclidean distances between neighboring digits were obtained. These distances represent the shortest path in 3D space, not the shortest path along the surface of the brain.

The absolute and relative spatial measures, being peak-vertex *ML (medial-lateral) x-axis coordinate* (for the right hemisphere obtained from the inverted left-right coordinate of the ACPC coordinate system), peak-vertex *AP (anterior-posterior) y-axis coordinate*, peak-vertex *IS (inferior-superior) z-axis coordinate*, and peak-vertex *distance to D1*, as well as the functionally related map measures, being *activation volume, peak value*, and *peak-vertex distance to neighbor digit*, were each statistically analyzed by two-way repeated-measures analysis of variance (RM ANOVA), using SPSS 24. The factors in the analysis were “digit” (5 levels: D1 to D5; for *distance to D1* and *distance to neighbor digit* only four levels: D2 to D5 and D1-to-D2 to D4-to-D5, respectively) and “side” (two levels: right dominant hand side and left non-dominant hand side). The main effects or interactions were declared significant if *p* < 0.05, with Greenhouse-Geiser corrections applied when necessary according to Mauchly's test of sphericity. Any significant main effects due to the factor “digit” were further explored by Bonferroni-corrected pairwise *post-hoc t*-tests between fingertips.

We further assessed the distance between the locations of the representations within subjects but across hemispheres and within hemispheres but across subjects. First, the Euclidean distance between the left-hand digit peak vertex and the mirrored (along the inter-hemispheric cleft) right-hand digit peak vertex of the corresponding digits of the right and left hand was calculated for each digit and subject; thereby, the average estimation error produced by mirroring across hemispheres within a subject could be calculated. Second, for each side and subject, the Euclidean distance between the subject's digit representation and the mean of the representations of the same digit in the same hemisphere for all other subjects was assessed; thereby, the average estimation error produced by averaging within a hemisphere across all other subjects could be calculated. The across-hemisphere within-subject and within-hemisphere across-subjects (for each subject averaged across the two sides) differences were compared using a two-way repeated measures ANOVA, with the factors “digit” and “method”. All results are reported as mean ± standard deviation.

## Results

fMRI measurements of sequential tactile stimulation of the first phalanx of the 5 fingertips were performed for the left and the right hand. The elicited BOLD responses in the contralateral primary somatosensory cortex were detected for each of the 10 digits in each of the 12 subjects. Extent of BOLD activation as well as *t*-values and coordinates of peak vertices within the activations were determined. The Euclidean representation distances from D1 to all other digits as well as between neighboring digits were calculated. Repeated measures ANOVAs were applied to identify differences of these measures across digits and sides. In addition to describing general similarities and differences between digits and left and right side, a separate analysis investigated the actual individual spatial distance between the within-subject position of measured left-hand and across-hemisphere mirrored right-hand digit representations and compared it to an alternative across-subject, within-hemisphere approach.

### Attention Task, Digit Succession

During the functional MRI runs, performed for the left and right hand separately, an average of 208 ± 5 interruptions were embedded in the sequential stimulation of the 5 fingertips, which the subjects were instructed to count. The mean difference between the presented and reported interruptions across sides and subjects was 5.4 ± 6.0%. This is a strong indicator that the subjects actively attended the stimulation.

**Figure 2:**
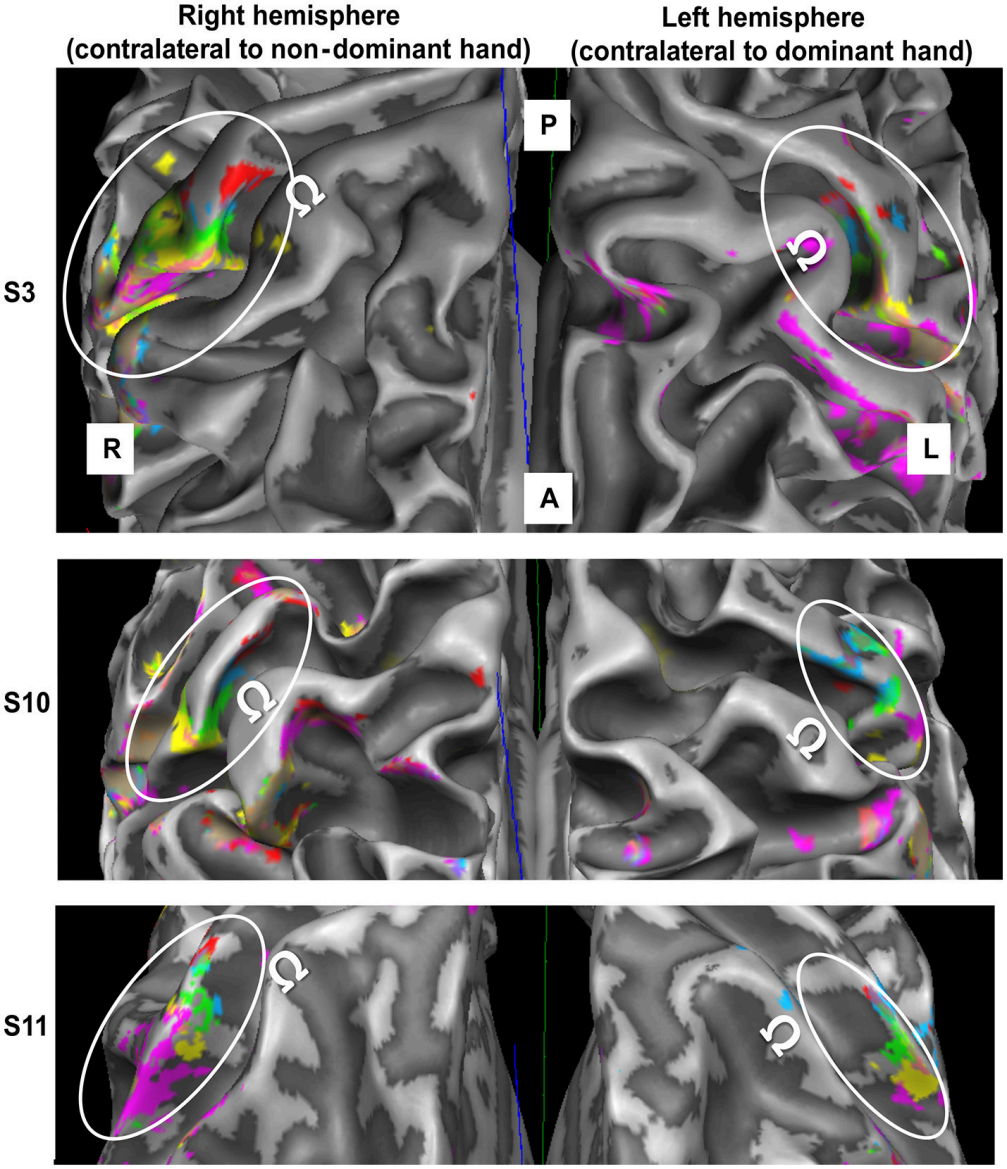
fMRI data obtained in the contralateral hemisphere in three subjects (top to bottom) through digit stimulation of the left hand (left panel) and right hand (right panel). For the third presented subject, the meshes were slightly inflated to improve the intrasulcal visibility (200 steps of inflation in Brain Voyager). A = anterior, P = posterior, R = right, L = left. White ellipses encircle the SI hand area. Ω defines the motor hand knob. Color code as in Figure 1.

One of the 12 subjects was excluded, as the left-hemispheric MR angiogram revealed a very large vessel running in parallel to the central sulcus, thereby preventing the observation of right-hand D1-to-D5 somatotopy. The mappings of the digits of both hands for this subject were excluded. For the remaining subjects, significant BA 3b activation was observed for each fingertip of each hand, not only at the volume level, but also after projection to the white-to-gray matter mesh and after vessel elimination. Figure 2 shows individual examples of the digit activations of both hands projected on the white-matter gray-matter surface reconstruction of the individual brains.

For both hands, visual inspection of the individual D5-to-D1 representations revealed the expected mediolateral digit succession along the central sulcus in BA 3b. The digit representations were always found in the same part of the post-central gyrus. For all subjects, a protrusion of the post-central gyrus was found slightly laterally to the precentral hand knob containing the motoric finger representation. The sensory D2 representation was usually very close to this protrusion, while D1 was represented slightly lateral to the latter. The D3 representation was almost always found at the rather straight part of the post-central gyrus medial to the protrusion, whereas D4 and D5 tended to be close to the medial anterior bend of the post-central gyrus.

### Activation Volume, Peak Vertex *t*-Value, Euclidean Distance to Neighbor Digit

**Figure 3:**
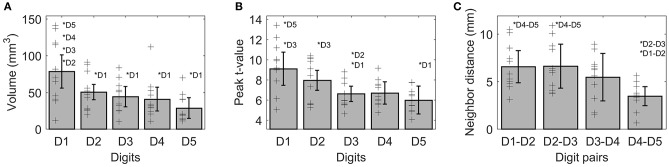
Visualization of the three functional measures, *volume* **(A)**, *peak t-value* **(B)**, and *neighbor distance* **(C)**. As no difference between sides was observed for any of these measures, the average ± standard deviation is shown as bar (across sides and subjects), individual subject results are represented as thin black crosses. Significant *post-hoc* tests are marked by asterisks.

On average (across sides, digits, and subjects), the activations elicited by the tactile stimulation of the digits had a size of 49 ± 37 vertices. The *volume* of the functional digit activations differed between the fingers [main effect “digit,” *F*_(10, 4)_ = 11.85, *p* < 0.001], with the largest activation volume for D1, as seen in Figure 3A. Statistically, a significant decrease in volume was observed from D1 to all other fingers. This difference in size of BOLD activation between fingers was comparable across the right and left side, since neither a significant main effect “side” nor an interaction “digit” x “side” could be observed (*p*-values given in Table 1).

**Table 1:** ANOVA results for all functional and structural measures.

**Functional map measures**	**Effect**	**df**	***F***	***p***
Volume	Digit	4	11.85	<0.001
	Side	1	< 0.01	0.97
	Digit × Side	4	2.54	0.113
Peak value	Digit	4	9.21	0.003
	Side	1	2.25	0.164
	Digit × Side	4	2.03	0.151
Distance to neighbor digit	Digit pair	3	4.64	0.009
	Side	1	0.46	0.511
	Digit pair × Side	3	0.232	0.873
Structural map measures	Effect	df	*F*	*p*
ML x-axis	Digit	4	35.95	<0.001
	Side	1	1.03	0.334
	Digit × Side	4	0.74	0.490
AP y-axis	Digit	4	45.96	<0.001
	Side	1	0.85	0.377
	Digit × Side	4	3.04	0.082
IS z-axis	Digit	4	32.51	<0.001
	Side	1	0.45	0.515
	Digit × Side	4	1.29	0.290
Distance to D1	Digit	3	57.03	<0.001
	Side	1	2.89	0.120
	Digit × Side	3	2.82	0.056

*Significant differences are shaded in dark gray, trends indicated in light gray*.

The average peak-vertex *t*-value across all digit activations had a level of 7.3 ± 2.5. The *peak values* also differed between the stimulated fingers [main effect “digit,” *F*_(10, 4)_ = 9.21, *p* = 0.003). While the *t*-value on average decreased from D1 to D5 (see Figure 3B), this effect was significant from D1 to D3 and D5 as well as from D2 to D3. No difference was obtained between the sides and no interaction was observed.

**Figure 4:**
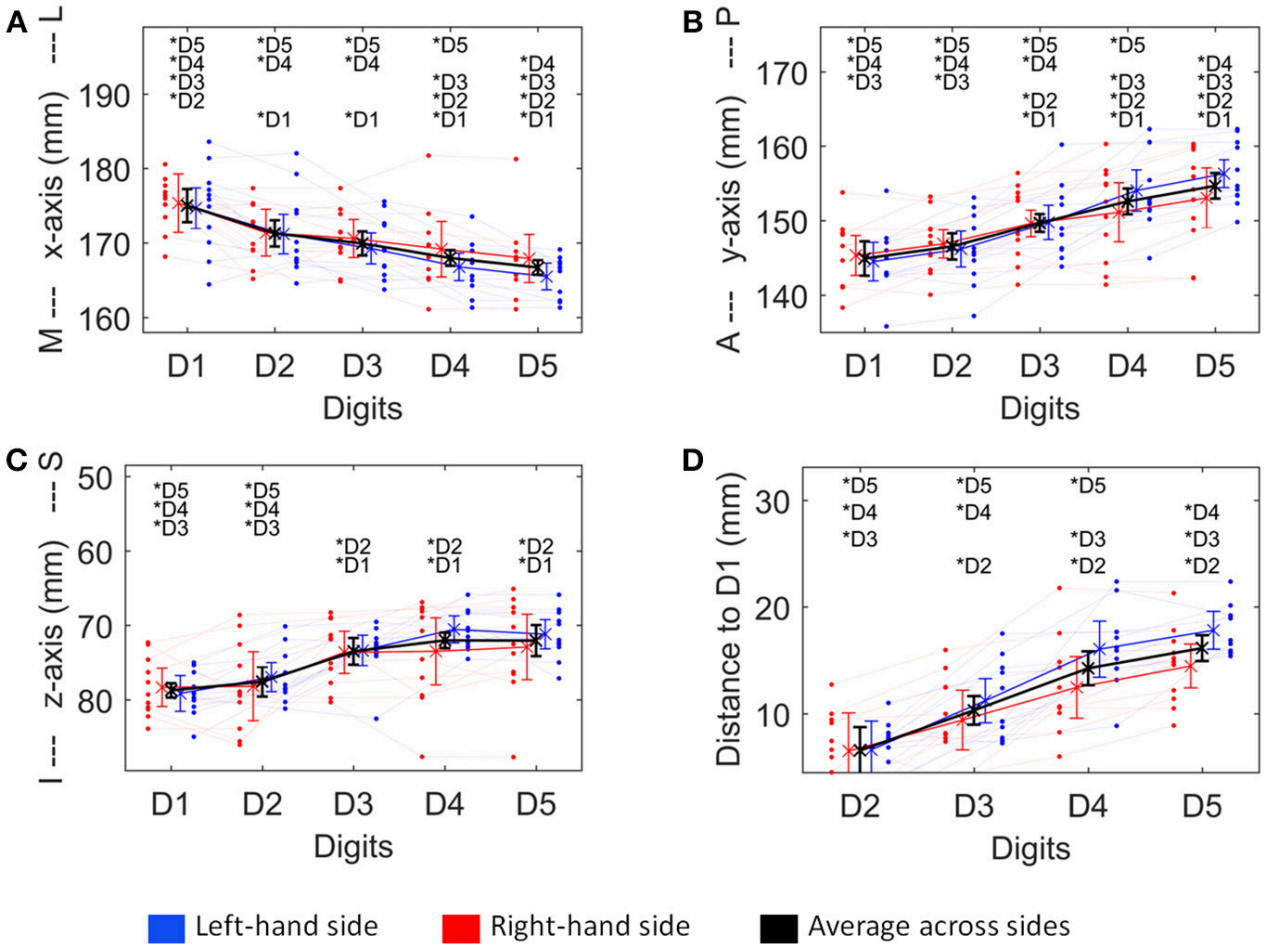
Visualization of the four structural measures, *ML x-axis* **(A)**, *AP y-axis* **(B)**, *IS z-axis* **(C)**, and *distance to D1* **(D)**. As a trend for a difference between sides was observed for some of these measures, not only the average ± standard deviation (across sides and subjects, thick black line), but also the average across subjects for the left (blue) and the right (red) hand digits, is shown. Individual subject results for each side are represented as thin lines in the respective color. Significant *post-hoc* tests between digits are marked by asterisks.

The Euclidean distance between neighboring digits (Figure 3C) differed between digit pairs (*F*_(10, 3)_ = 4.64, *p* = 0.009); for the D1-D2 and D2-D3 distance vs. the D4-D5 distance the *post-hoc* tests were significant. Neither a significant main effect of “side” nor a significant interaction was observed. The summed Euclidean distance between the neighboring digits D1-D2 through D4-D5 was 23 ± 4 mm for the left and 21 ± 8 mm for the right hand. As a second Euclidean measure, the distance to D1 was calculated for D2 through D5 and then compared between digits and sides. This analysis also revealed a main effect of “digit,” reflected by a general increase in distance to the thumb from D2 to D5 (see Figure 4D). All *post-hoc* tests were significant, confirming the main effect. While the main effect of “side” was non-significant, a strong interaction trend was found (*F*_(10, 3)_ = 2.82, *p* = 0.056), with the left-side D1-to-D4 and D1-to-D5 distance tending to be larger than the right-side D1-to-D4 and D1-to-D5 distance. The direct D1-D5 Euclidean distances (left hand digits = 18 ± 2 mm, right hand digits = 14 ± 4 mm) were, as expected, considerably shorter than the summed Euclidean neighboring digit-pair distances.

### Peak-Vertex Coordinates

The absolute spatial positions of the peak vertices of all ten digit activations along the x-, y-, and z-axis were explored after mirroring the left-hemisphere digit peak-vertex position onto the right hemisphere, meaning that the new x-coordinate was equally far from the hemispheric cleft in the right hemisphere as it was in the left hemisphere before. A significant main effect of the factor “digit” [*p* < 0.001, *F*_(10, 4)−_values see Table 1] was observed for all three AC-PC coordinate axes (*ML x-axis coordinate, AP y-axis coordinate, IS z-axis coordinate*), reflected by the representation of D1 being most lateral-anterior-inferior to D5 being most medial-posterior-superior and each digit having a distinct position in this coordinate system (see Figures 4A–C). The *post-hoc* tests corroborated this observation for each coordinate, as significant differences were observed in 24 out of the 30 digit comparisons (except for the D2 to D3 along the x-axis, D1 to D2 along the y-axis, and D1 to D2 as well as D3 to D4, D3 to D5, and D4 to D5 along the z-axis).

In contrast, there was no significant main effect for the factor “side” in any coordinate, indicating no consistent difference in the position of the averaged digit representations across subjects between the left and the right hand, i.e., digit representations were not generally shifted into one direction from one hemisphere to the other. The only indication of a possible difference between the right and the left hand digits was a trend in the interaction between “digit” and “side” along the anterior-posterior y-axis [*F*_(10, 4)_ = 3.04, *p* = 0.082], in particular due to the left-hand D4 and D5 representations being positioned more posterior than the right-hand D4 and D5 (Figure 4B), which parallels the trend in larger left-hand D1-D4 and D1-D5 Euclidean distances. At the x- and z-axis no trend or significant interaction between “side” and “digit” was observed.

### Distances Between Observed And Estimated Digit Representation Locations

**Figure 5:**
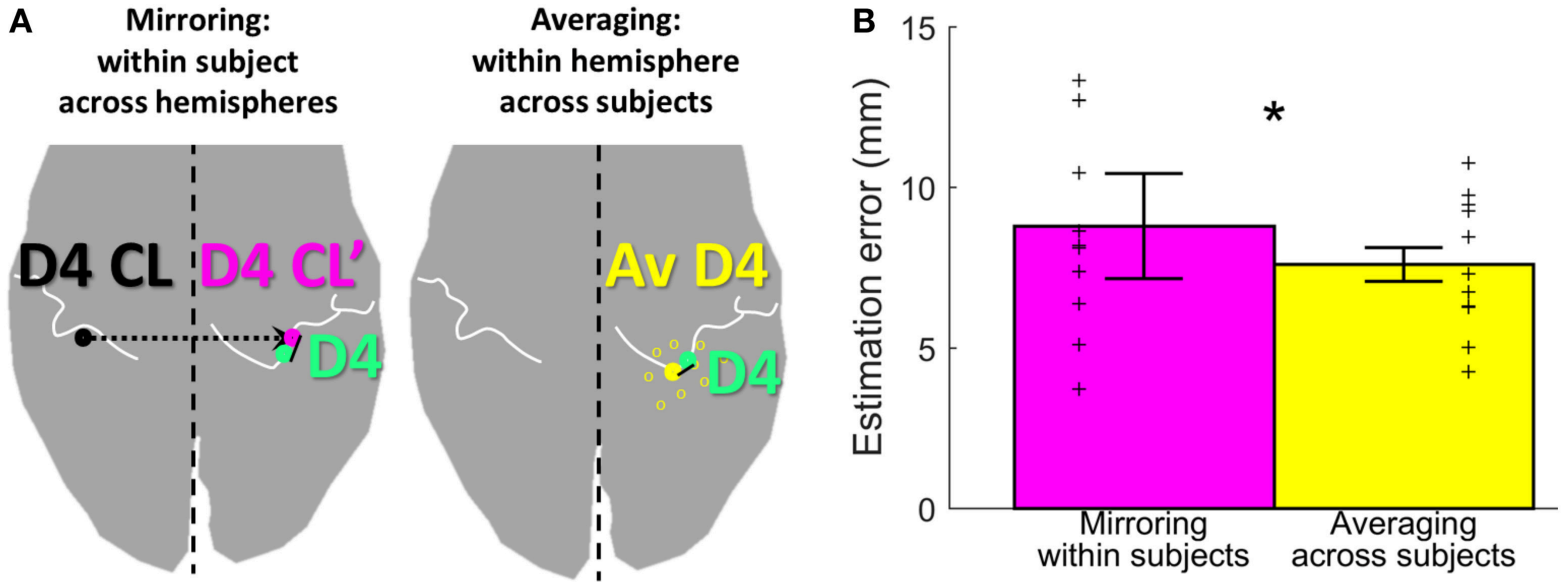
Estimation methods and results. **(A)** Visualization of the two estimation methods mirroring (left, in pink) and averaging (right, in yellow), for the example of D4 estimation (measured D4 shown in green). **(B)** Visualization of the estimation error, shown for each of the two estimation methods (mirroring in pink and averaging in yellow) as mean ± standard deviation, averaged across subjects and digits. Individual subject results for each method are represented as thin crosses. The significant main effect is marked by an asterisk.

To assess the offset between the finger representations of the two hemispheres in each subject, the Euclidean distance between the individual left-hand peak vertex and the respective mirrored (along the inter-hemispheric cleft) individual right-hand peak vertex of each digit was calculated. On average (across subjects and digits), the distance between the true and the within-subject, across-hemispheres mirrored representation was 8.8 ± 4.6 mm (Figure 5, pink bar). For the individual digits, the distances were 7.3 ± 2.7 mm (D1), 8.1 ± 5.1 mm (D2), 7.7 ± 3.6 mm (D3), 11.3 ± 5.5 mm (D4), and 9.6 ± 5.1 mm (D5).

As an alternative approach to estimate digit representations, not the individual digits were mirrored, but an average of the same hemispheres' digit positions across the other measured subjects was compared to the individual digit representation. On average across subjects, sides, and digits, the distance between the subject's and the across-subjects (within-hemisphere) averaged representations was 7.6 ± 2.7 mm (Figure 5, yellow bar). For the individual digits, the distances were 7.1 ± 2.4 mm (D1), 7.8 ± 3.2 mm (D2), 7.1 ± 2.6 mm (D3), 8.4 ± 2.2 mm (D4), and 7.7 ± 3.4 mm (D5). The average distance across digits obtained with the across-subjects within-hemisphere approach was significantly smaller than the distance obtained by within-subject, across-hemisphere mirroring (main effect “method,” *F*_(10, 1)_ = 8.05, *p* = 0.018], with no significant difference between digits [F_(10, 4)_ = 0.64, *p* = 0.638] and no interaction between “digits” and “method” [*F*_(10, 4)_ = 1.00, *p* = 0.4212]. This indicates that the distance between the actual and the estimated locations of digit representations was on average 1.2 mm smaller when the unrelated group average of the same hemisphere was used than when the own individual digit location from the other hemisphere was mirrored.

## Discussion

The comparison of the BOLD activation elicited by tactile stimulation of the ten fingers of the dominant and non-dominant hand showed a main effect “digit” for each of the analyzed measures, specifying that they are not only distinct entities but also differ in their extent of functionally and structurally related measures. This was in contrast to the main effect “side,” where no general difference between the digit representation of the right and the left hand could be determined in any of the measures. The same holds true for the interaction between side and digit, where no significant difference was detected, indicating a similar general layout of the left-hand and right-hand digit maps across subjects. The only sign of a deviation concerning the side-digit interaction was a non-significant trend that pointed to a larger cortical D1-D4 and D1-D5 spread for the left hand, which was paralleled by a trend of a more posterior position of the left ring and little finger representation (D4, D5). Despite the general correspondence of the position of the digit representations between the two hemispheres across subjects, considerable prediction errors emerged when estimating an individual's digit location through within-subject, across-hemispheres mirroring of the homologous digit position. Based on the above results the error could be slightly reduced by using the across-subjects, within-hemisphere average of the respective digit as an estimator.

Many studies have explored and described the fMRI fingertip somatotopy within a single hemisphere using the peak-voxel approach (e.g., Nelson and Chen, 2008; Schweizer et al., 2008; Stringer et al., 2011), consistently demonstrating that the digit representations form an organized somatotopic BA 3b map along the posterior wall of the central sulcus. In the present study, the more functionally related measures showed that the single digit representations differ in several aspects. Most prominently, the thumb representations had larger volumes and together with the index-finger representations higher activation and a larger distance to the neighboring digits. This is probably explained by the higher importance and the more extensive usage of the thumb and index finger in tactile sensing. The absolute spatial coordinates further support this lateral-anterior-inferior to medial-posterior-superior digit somatotopy for both hands. In particular, the somatosensory digit representations were found in the same area of SI for all subjects. This area can be defined based on the next anterior protrusion of the post-central gyrus lateral to the precentral motor hand-knob location (Yousry et al., 1997). The D1, D2, and D3 to D5 representations were usually located lateral, close to, and medial to this landmark.

No statistical differences were observed between the left and right hand digit representations in the more functionally related measures of volume, activation strength, and relative distance to neighboring digit representations. Specifically the Euclidean distances between neighboring digits, being very local and with minimal structural influence, can be seen as indicators for the layout of the functional digit map, showing no difference between the right dominant and left non-dominant hand. Hence, the dominant hand does not have stronger or larger tactile related digit representations compared to the non-dominant hand. This finding is in accordance with the fact that also the tactile measure of fingertip acuity does not seem to depend on the handedness (Sathian and Zangaladze, 1996).

In contrast to the just discussed more functionally related measures, the absolute x-, y-, and z-axis coordinates represent both functionally related aspects determined by the layout of the functional map as well as structural aspects related to the anatomical layout of the sensory digit area at the post-central gyrus on which the functional map is superimposed. Since the more functionally related measures, particularly the relative distance between neighboring digit representations, revealed no difference between the dominant and the non-dominant hand, the layouts of the functional digit maps of both hands are assumed to be comparable across subjects. Also for the absolute position of the digit representations along the medial-lateral x-, the superior-inferior z-, and the anterior-posterior y-axis no significant side-related differences were observed, further corroborating the general similarity of the location of the digit representations of the two hands across subjects.

Still, two related, non-significant trends were observed, being the more posteriorly located left-hand D4 and D5 digit representations and the larger Euclidean distances of these two representations to the D1 representation, which could be interpreted as slight evidence for more structurally related differences between the left- and right-hand digit representations. There are reports of structural asymmetries of the hemispheres with a leftward tendency of sulcus depth in right-handers (Maingault et al., 2016), larger left-hemispherical regional asymmetry in gyrification in subjects with strong right-handedness (McDowell et al., 2016) as well as a shape difference in the hand-knob area between the hemispheres with the greatest difference observed in right-handers (Sun et al., 2012). However, due to the limited sample size and the absence of a detailed structural analysis, the present data do not allow for general conclusions about structural differences in the central-sulcus hand area between hemispheres across subjects. Still, structural variations could be one factor to explain the rather large spatial variations between homologous digits within subjects discussed below.

The comparison of the spatial position of homologous digit representations in the two hemispheres has clinical implications in unilateral transradial or transhumeral amputees, in which the sensorimotor incongruence between perceived phantom movements and the missing expected sensory response is one of the most discussed mechanisms responsible for phantom-limb pain, possibly resulting in maladaptive cortical plasticity and a smaller representation area for the amputated digits, hand, or arm (Flor et al., 1995; Lotze et al., 1999, 2001). In these amputees, the degree of reorganization of the functional maps after amputation has been estimated by determining the hand area from the unaffected side and mirroring the position onto the affected side (Flor et al., 1995). This is based on the assumption that the structural layout, as well as the functional maps, are comparable in the two hemispheres. The results of the present study show that both assumptions are met at the across-subject level: no statistical differences between the location of the digit representations of the right and left hands were observed across the analyzed measures. The mirroring approach is, however, applied in single subjects. There, the within-subject estimation error is relevant, and the current data revealed a distance of around 9 mm between the mirrored and the measured homologous digit representation, well-above the Euclidean distance between neighboring digits. Since the only two measures showing at least a trend for a difference between the right- and the left-hand digit representations across subjects were rather of structural than of functional nature, the influence of structural variations of the central sulcus digit areas between hemispheres could be a factor contributing to the estimation error. Hence, in a subject with a unilateral single-digit amputation, that digit's representation location prior to amputation could only very roughly be estimated with this method. For these amputees, the comparison of the Euclidean distances between two, ideally rather close digits on one vs. the other hand (Weiss et al., 1998) is favorable over mirroring, since this measure is short-range and more independent from the spatial locations and the anatomy of the central sulcus. However, in the case of unilateral hand or arm amputations, the mirroring approach can, e.g., for the original magnetoencephalography studies, be justified by the large shifts of the adjacent SI chin area into the SI digit area at the amputated side [(Elbert et al., 1994): 5–33 mm; (Yang et al., 1994): 30-35 mm, (Flor et al., 1995): 5–45 mm], which are predominantly beyond the here obtained digit estimation error caused by mirroring. Hence, the described mirroring approach can be used in these cases to explore whether maladaptive plasticity is present and whether an approach for phantom-limb pain reduction managed to partly redo the shift.

Since the present analysis indicates that the structural differences between the hemispheres could be a confounding factor in mirroring, an alternative for the single-subject estimation approach was implemented using the average digit coordinates (across subjects) from the same hemisphere. The same hemisphere was chosen to reduce the influence of differences in the anatomical layout of the central sulcus in the two hemispheres (Sun et al., 2012). The significantly reduced estimation error of this within-hemisphere, across-subjects approach confirms the error-reducing effect of using the averaged location of digit representations from the same hemisphere. This can especially be seen at the digits D4 and D5 (statistical trend) in comparison to the other digits, which confirms the influence of the structural differences between the hemispheres on the mirroring approach. Hence, the SI digit locations of a healthy, age-, gender-, and handedness-matched reference group could be an alternative approach to predict the pre-amputational sensory digit area in unilateral amputees or to explore whether phantom-finger activations elicited by stimulation of the stump are in the range of the normal sensory digit area, as done previously by Björkman et al. (2012). However, this approach again does not provide sufficient accuracy for the prediction of the spatial position of single digit locations, due to the still considerable estimation error of around 8 mm resulting from structural and functional variability between subjects.

The clinical relevance of this study is not limited to amputees. During recovery after stroke, a posterior shift (Pineiro et al., 2001) as well as an extension into the face area (Weiller et al., 1993) have been observed in the primary sensorimotor cortex. Since the activation pattern of the non-affected hand (in the hemisphere ipsilateral to the affected hand) was also found to be altered (Nair et al., 2007), a comparison to an adequate reference group instead of the same patient's other side may be preferable. Significant shifts in the primary sensorimotor area were also observed in multiple-sclerosis patients (Lee et al., 2000) as well as in patients with a brain tumor in the motor cortex (Seitz et al., 1995). In all these cases where no digit representation of the opposite hemisphere can be obtained a database of adequately-matched control samples or mirrorring can be used as an approximate estimation, always considering the limits of the two approaches.

In conclusion, this study shows that the functional maps of the digit representations of both the dominant (right) and the non-dominant (left) hand follow a well-defined lateral-anterior-inferior to medial-posterior-superior pattern, consistently located around a protrusion in the post-central gyrus. The digit representations of the right, dominant and the left, non-dominant hand did not differ in volume, strength, or distance of digit representation to neighboring digits, indicating a similar layout of the functional digit maps of the two hands across subjects. Also, no significant differences in x-, y-, or z-axis position of the digit-representation locations were observed between the right and left hands, indicating no consistent structural variations in the hemispheres across subjects. On the clinically relevant single-subject level rather large differences in spatial position between homologous digit representations were obtained. These estimation errors, potentially caused by small differences in the individual structural layout of the central-sulcus hand area should be taken into account when trying to predict a missing digit representation of a patient. Alternatively or when no reliable digit representation location can be obtained from neither hemisphere, the present data can be used as a within-hemisphere, across-subjects reference for right-handed, same-age subjects in pathological conditions, probably yielding a slightly smaller but still considerable prediction error.

## Author Contributions

MS, RS, and JF designed the experiment. MS conducted the experiment and analyzed the data. MS and RS wrote the paper, all authors contributed to manuscript completion.

### Conflict of Interest Statement

The authors declare that the research was conducted in the absence of any commercial or financial relationships that could be construed as a potential conflict of interest.
